# Valuation of Endowment-Insurance Equity-Linked Contracts for Stocks with Exotic Dynamics

**DOI:** 10.1155/2014/314286

**Published:** 2014-02-11

**Authors:** Javier Villarroel

**Affiliations:** Facultad de Ciencias, Universidad de Salamanca, Plaza Merced, 37008 Salamanca, Spain

## Abstract

We consider the fair martingale prize of insurance contracts with benefit received either at the insurer's demise or at maturity. We show how to modify the dynamics of the underlying so as to incorporate the possibility that the traded stock has a strong support at some level. The resulting dynamics is integrated and the fair prize of several natural endowment-insurance contracts is obtained.

## 1. Introduction

In this paper we consider some explicit formulas regarding the valuation of a certain class of equity-linked contracts with a general premium, the endowment-insurance policies. As it is well known an equity-linked contract is a life insurance product where the benefit depends upon the value of some reference equity fund or portfolio which is traded in some associated market. In addition, most unit-linked contracts guarantee a minimum amount if the stock price falls below a fixed level. The pricing of equity-linked policies is a classical problem in the actuarial literature first discussed by Brennan and Schwartz [1] and Boyle and Schwartz [2]. See also Bacinello and Ortu [3], Aase and Persson [4], Brennan and Schwartz [5], Ekern and Persson [6], Boyle and Hardy [7], Grosen and Jorgensen [8], Moeller [9], Bernard et al. [10], Bacinello [11], and Shen and Xu [12].

The fair value of these kinds of products involves considering two separate sources of randomness: one stemming from the stochastic nature of the dynamics of the stock markets and a different one due to the uncertainty in mortality. Here we consider a simple model of financial market consisting of two securities: a savings account *B*_*t*_ which evolves via *dB*_*t*_ = *r*_*t*_ *B*_*t*_ *dt*, where *r*_*t*_ is the instantaneous interest rate of the market. For convenience it is assumed to be deterministic, but not necessarily constant as befits a contract held for a long time. The second instrument in the market, to which the policy is linked, is a given stock whose *t*-price, *X*_*t*_, varies according to definite stochastic dynamics. The prototype model for stocks-price evolution [13, 14] assumes that *X*_*t*_ is a geometric Brownian motion (GBM) model; that is, *X*_*t*_ satisfies the stochastic differential equation (SDE)

(1) $$\begin{array}{c} dX_{t}=\mu X_{t}dt+\sigma X_{t}dW_{t}. \end{array}$$

Here *μ* is the mean return rate and *σ* is the volatility, while *W*_*t*_ is a Brownian motion under the empirical or real world probability. Despite the fact that this simple model describes well the basic dynamical properties, there are nonetheless several stylized facts that the model fails to capture.

In this paper we modify the dynamics so as to incorporate the possibility that the traded stock has a strong support at some level, say *c*. Such a feature may stem from a market consensus under which heavy buy orders are triggered when the stock price hits this level. Investing in such stock may well be an appealing feature to the insurance company as it partially hedges the exposure to a possible plummeting of the price. In Section 4 we propose a dynamical model which incorporates such a feature by modifying appropriately the “infinitesimal volatility” term; by using Feller's theory, we discuss the nature of the barrier. We next show that under some election of parameters the SDE that drives the dynamics can be solved in an exact way (see (39) to (42)). We suppose that the benefit is received either at the insurer's demise or at maturity, whichever comes first.

The structure of the paper is the following. In Section 2 we give the basic setup and determine explicit formulas for the fair premium required by the insurance firm to hedge the exposure to the evolution of the risky asset for an endowment insurance contract under quite general conditions. The fair value is given in terms of expectations with respect to the risk-free measure under which the discounted price of any security happens to be a martingale. In Section 3, assuming a simple GBM dynamics, concrete valuation formulas for several contracts of actuarial interest are obtained (see formulae (9)-(10), (17)–(20), and (26)-(28)). Recall that the classical work of Brennan and Schwartz [1] and Boyle and Schwartz [2] corresponds to a pure endowment contract with payoff at maturity Ψ_*T*_ ≡ max⁡{*X*_*T*_, *k*}. Remarkably, when the guaranteed amount is taken as the interest accrued with rate *δ* for a principal *X*_*t*_, then the premium contingent on death can also be obtained in closed form, as we show below. The second case we study corresponds to a floating strike lookback put (FLOP) which entitles the owner to sell the stock at the highest realized price, that is, to a path dependent payoff Ψ_*T*_^1)^ = **X**_*T*_^*t*^ − *X*_*T*_, where **X**_*T*_^*t*^ ≡ max⁡_*t*≤*s*≤*T*_ *X*_*s*_ is the running maxima of the process. Lookback options guarantee a nonloss outcome and as such are interesting in dynamic investment fund protection.

In Section 4 we obtain and solve a natural stochastic equation that models evolution in a situation when strong resistances are present (see (39) to (42)), whereupon we obtain the fair insurer's liability for several endowment-insurance contracts. The presence of barriers is found to complicate the prizing problem.

Section 5 is devoted to study the partial differential equation (PDE) that the premium satisfies. We derive under appropriate conditions a modified Kolmogorov Backwards equation (68) and sketch a brief comparison between replicating portfolios techniques and direct martingale approaches.

## 2. Contract Characteristics and Valuation

As we have already pointed out, unit-linked contracts involve two sources of randomness, one stemming from the associated financial market and another corresponding to mortality expectations. We now pass to discuss some features of the latter. Consider a given individual aged *d* at time 0 and let $\tau :\bar{\Omega }\to \mathbb{R}$ be the time at which decease occurs. Here, *τ* is a random variable defined on a complete probability space $\left(\bar{\Omega },\bar{𝒢},\bar{\mathbb{P}}\right)$. Under natural assumptions the survival function ${}_{\hat{T}}p_{t+d}$ is given by

(2) $$\begin{array}{c} {}_{\hat{T}}p_{t+d}\equiv \overset{-}{\mathbb{P}}\left(\tau >T\;|\;\tau >t\right)=e^{-\overset{T}{\underset{t}{\int }}\mu (d+s;s)ds}\equiv \overset{\infty }{\underset{T}{\int }}h\left(s\right)ds, \end{array}$$

where *t* ↦ *h*(*T*) the conditional density of *τ* and $\hat{T}\equiv T-t$. Further the mortality intensity is taken in the Gompertz-Makeham form *μ*(*t* + *d*, *t*) = *α* + *βλ* ^(*t*+*d*)^ (see [15, 16]). We finally introduce the filtration ${\bar{ℱ}}_{t}\equiv \sigma (1_{\left\{\tau >s\right\}},\;0\leq s\leq t)$.

In a unit-linked contract the premium *v*_*t*_ paid at time *t* by policyholders is invested in a equity fund. Let *X*_*t*_ be the value at time *t* of a unit of the fund and (*Ω*, *ℱ*_*∞*_, *ℙ*) be a probability space big enough to contain the filtration *ℱ*_*s*_ generated by the stock: *ℱ*_*t*_ ≡ *σ*(*X*_*s*_,  0 ≤ *s* ≤ *t*). Let Ψ_*T*_^1)^ be the reward payable at maturity *T* if the policyholder is alive. Further, if the insurer's demise happens at a time *τ* before maturity, the policy entitles the beneficiaries to a payment Ψ_*τ*_^2)^ at the decease time. In other words, we consider an endowment insurance, mixing a pure endowment and a term insurance contract. Here Ψ^1,2)^ are supposed to be right continuous processes adapted to the filtration *ℱ*_*s*_. These conditions guarantee that Ψ^1,2)^ are progressively measurable and that Ψ_*τ*_^2)^ is *ℱ*_*τ*_ measurable. Note that in our basic setup surrender is not permitted; however, it could be easily incorporated by treating the surrender decision like a premature decease, as several authors do.

We assume that the insurance company is risk-neutral with respect to mortality. This assumption means that it does not request any compensation for assuming mortality risk. The exposure could be hedged to some extent whenever a large number of contracts are written yearly.

We will also assume that our market is efficient; that is, the existence of the Harrison and Pliska [17] risk-neutral probability on (*Ω*, *ℱ*_*∞*_) under which discounted prices of self-financing strategies *v*_*t*_′ ≡ *v*_*t*_/*B*_*t*_ are martingales with respect to the history of the process up to time *t*.

The pricing of equity-linked life insurance policies is a classical problem in the actuarial literature and, particularly for pure endowment contracts, explicit formulas are known. However, far less is known as regards explicit analytical formulas for endowment insurance contracts even for the simplest GBM dynamics. Here we determine the insurer's liability required by the insurance firm to hedge the payoff for given Ψ^1)^, Ψ^2)^, and a general mortality intensity *μ*(*d* + *t*, *t*).

Let *v*_*t*_ be the price of such a contract written at time *t* at which *X*_*t*_ ≡ *x* is known and maturing at time *T*. Let $𝔼\equiv {𝔼}^{\mathbb{P}\times \bar{\mathbb{P}}}$ denote the expectation with respect to the product measure $\mathbb{P}\times \bar{\mathbb{P}}$. Let ∂ be the demise time or maturity, whichever comes first, ∂ = *T*∧*τ*. Then, we have

(3) vt′=𝔼(v∂′ ∣ ℱt∨ℱ¯t)=𝔼(vT′1{τ≥T}+vt′1{t≤τ<T} ∣ ℱt∨ℱ¯t)=𝔼(Θ ∣ ℱt∨ℱ¯t).

Here *v*_*t*_′ ≡ *v*_*t*_/*B*_*t*_ are the deflated prices and we used the martingale property and the optional stopping theorem (note that the optional stopping theorem can be applied since $\bar{\mathbb{P}}(\partial <\infty )=1$, *𝔼* ^*ℙ*^(sup⁡_0≤*s*≤*T*_Ψ_*s*_^*i*)^) < *∞*). Further,

(4) $$\begin{array}{c} \Theta \equiv \frac{\Psi_{T}^{1)}}{B_{T}}1_{\left\{\tau \geq T\right\}}+\frac{\Psi_{\tau }^{2)}}{B_{\tau }}1_{\left\{t\leq \tau <T\right\}} \end{array}$$

is a function naturally defined on the product probability space $\left(\Omega \times \bar{\Omega },{ℱ}_{\infty }\vee {\bar{ℱ}}_{\infty },\mathbb{P}\times \bar{\mathbb{P}}\right)$, adapted to the augmented filtration: ${ℱ}_{t}\vee {\bar{ℱ}}_{t}\equiv \sigma ({ℱ}_{t}\cup {\bar{ℱ}}_{t})$. Equation (2) involves

(5) 𝔼(ΨT1)1{τ≥T} ∣ ℱt∨ℱ¯t)=𝔼ℙ¯(1{τ≥T} ∣ ℱ¯t)𝔼ℙ(ΨT1) ∣ ℱt)=T^pd+t1{t≤τ}𝔼ℙ(ΨT1) ∣ ℱt),

where we assume that *ℱ*_*t*_, ${\bar{ℱ}}_{t}$ are independent filtrations, as it is reasonable to assume that the risk stemming from the market has no influence on the mortality risk.

Similarly we have in terms of the conditional density *h* of *τ* (see (2))

(6) 𝔼(1{t≤τ<T}Ψτ2)Bτ ∣ ℱt∨ℱ¯t)  =𝔼[𝔼(1{t≤τ<T}Ψτ2)Bτ ∣ ℱt∨ℱ¯t,τ) ∣ ℱt∨ℱ¯t]  =1{t≤τ}𝔼(∫tTdsh(s)BsΨs2) ∣ ℱt∨ℱ¯t)    =1{t≤τ}𝔼ℙ(∫tTdsh(s)BsΨs2) ∣ ℱt).

Thus the insurer's liability at time *t* is made up of two terms, in correspondence with the benefits at maturity or at decease:

(7) $$\begin{array}{c} v_{t}={}_{\hat{T}}p_{d+t}v_{t}^{1)}+v_{t}^{2)}, \end{array}$$

where

(8) vt1)=1{t≤τ}BtBT𝔼ℙ(ΨT1) ∣ ℱt),vt2)≡1{t≤τ}𝔼ℙ(∫tTdsh(s)BtBsΨs2) ∣ ℱt).

For obvious reasons, the term 1_{*t*≤*τ*}_ will be dropped in the sequel. For guaranteed unit-linked contracts (GULC) the benefit at maturity depends on the value of the associated portfolio but there is a minimum guaranteed amount if the stock price falls below a fixed level; this can be taken to correspond to the capital accrued at a fixed interest rate *δ*, the “technical rate.” A fairly common example (see, [12]) is given by the choice Ψ_∂_^1,2)^ = *X*_∂_ + *X*_*t*_(*e* ^*δ*(∂−*t*)^ − 1), where contingent on an insurance event happening (maturity ∂ = *T* or death ∂ = *τ*) the insured receives the stock plus the interest accrued with rate *δ*. It is remarkable that in such situation it is possible to derive a closed expression for the fair value with all generality, independently of the dynamics of the stock. Indeed, using that *X*_*t*_/*B*_*t*_ is a martingale we find that the maturity and mortality premiums for such a GULC are given by

(9) vt1)=Xt(1+BtBT(eδT^−1)),vt2)=Xt(1−T^pd+t+∫tTdsh(s)BtBs(eδ(s−t)−1)).

When the interest and mortality rates *r* and *μ* are constants and *X*_*t*_ is assumed to have standard Black and Scholes dynamics one recovers the result of Shen and Xu [12] obtained by PDE techniques.

In a generic case, explicit evaluation of *v*_*t*_^1)^ and *v*_*t*_^2)^ can be a difficult matter. Those cases when Ψ_*t*_^2)^ is either a Markov process with a time homogeneous transition function or when it can be represented as Ψ_*τ*_^2)^ = Ψ_2_(*τ*, *X*_*τ*_) in terms of a given measurable function Ψ_2_ : ℝ^2^ → ℝ are of particular interest; in this case *v*_*t*_^2)^ can be simplified as follows. Let *θ*_*s*_ be the standard time-shift map on the path space acting on sample paths *ω* ∈ *Ω* via (*θ*_*s*_∘*ω*)(*t*) = *ω*(*t* + *s*). Then, Markov's property yields

(10) vt2)≡𝔼ℙ(Bt∫tTdsh(s)BsΨs2) ∣ ℱt)=𝔼ℙ(Bt∫0T^h(l+t)Bl+tΨl+t2)dl ∣ ℱt)=𝔼ℙ(Bt∫0T^h(l+t)Bl+t(θt∘Ψl2)) ∣ ℱt)=[∫0T^h(l+t)Bl𝔼xℙΨl2)dl]x=Xt.

## 3. Valuation of GUL in a Black and Scholes Framework

### 3.1. Endowment Insurance Contracts

In the sequel we study the valuation of two different types of GULC of actuarial interest. The first case generalizes the result of Brennan and Schwartz [1] and Boyle and Schwartz [2] from a pure endowment case to an endowment insurance contract, where the payoff contingent on death occurring before maturity is given by Ψ_*τ*_^2)^ = max⁡{*X*_*τ*_, *X*_*t*_ *e* ^*δ*(*τ*−*t*)^}. This means that the initial capital, accrued at a fixed interest rate *δ*, is guaranteed. We also study the nonarbitrage price for a floating strike look-back put (FLOP) which entitles the owner to sell the stock at the highest realized price before maturity, that is, to a terminal payoff Ψ_*T*_^1)^ = **X**_*T*_^*t*^, where **X**_*T*_^*t*^ ≡ max⁡_*t*≤*s*≤*T*_ *X*_*s*_ is the running maxima of the process. Lookback options guarantee a nonloss outcome and as such are interesting in dynamic investment fund protection.

In this section we assume classical stochastic dynamics so that under the martingale probability *X*_*t*_ evolves via a geometric Brownian motion. Let *T* ↦ *X*_*T*_^*t*,*x*^ be the solution that starts from *x* at time *t*, *t* < *T*:

(11) $$\begin{array}{c} X_{T}^{t,x}=X_{t}\exp\left\{\sigma \left(W_{T}-W_{t}\right)+\overset{T}{\underset{t}{\int }}r_{s}ds-\frac{\sigma^{2}\left(T-t\right)}{2}\right\}, \end{array}$$

where we allow for a time varying short term interest rate *r*_*t*_, since a life insurance policy could typically be expected to be held for a long time. However, to obtain closed formulas for the premium contingent on death *v*_*t*_^2)^ we assume in this section that *r* is constant.

The premium for a contract with payoff Ψ_*T*_^2)^ = max⁡{*X*_*T*_, *X*_*t*_ *e* ^*δ*(*T*−*t*)^} is written at time *t* such that *X*_*t*_ = *x* follows a minor modification of the classical Black-Scholes-Merton formula: the equality in law $X_{T}^{t,x}=X_{\hat{T}}^{0,x}$, where $\hat{T}=T-t$ yields that *v*_*t*_^1)^ ≡ *v* ^1)^(*T* | *t*, *x*) = *v* ^1)^(*T* − *t* | 0, *x*), where

(12) $$\begin{array}{c} v^{1)}\left(T\;|\;0,x\right)=x\left(\Phi \left(m_{+}\sqrt{T}\right)+e^{-\hat{r}T}\Phi \left(-m_{-}\sqrt{T}\right)\right), \end{array}$$

Φ is the normal distribution function, and

(13) $$\begin{array}{c} m_{\pm }\equiv \frac{\hat{r}}{\sigma }\pm \frac{\sigma }{2},\;\hat{r}\equiv r-\delta . \end{array}$$

The limit behavior for long values of *T* is interesting; depending on whether $\hat{r}>0$, $\hat{r}=0$, or $\hat{r}<0$, three different possibilities are found for the premium: *v* ^1)^(*T* | *t*, *x*) tends, respectively, to either *x* ≡ *v* ^1)^(*t* | *t*, *x*), to 2*x*, or to *∞*. The result is easy to understand. The higher the guaranteed rate the more interesting the contract becomes. Further, when *δ* is higher than the market rate *r*, the discounted benefit tends to *∞* asymptotically in time and so does the premium.

As commented, it turns out that an analytical formula can be derived for the demise component premium when *r* and *μ* are independent of time. Here Ψ_*τ*_^2)^ = max⁡{*X*_*τ*_, *X*_*t*_ *e* ^*δ*(*τ*−*t*)^}. In this case, using (10) we find that *v* ^2)^(*T* | *t*, *x*) = *v* ^2)^(*T* − *t* | 0, *x*), where

(14) v2)(T−t ∣ 0,x)  =xμ∫0T(e−μsΦ(m+s)+e−(μ+r^)sΦ(−m−s))ds

and hence it involves the integral

(15) I≡∫0Tdse−αsΦ(ms)≡∫0Tdse−αs∫−∞mse−z2/2dz2π.

By interchanging integrals we find *I* to be given by

(16) I=12α[1−|m|m2+2α+2|m|m2+2αΦ(|m|m(m2+2α)T)    −2e−αTΦ(mT)].

It follows that the demise contribution to the premium is given by

(17) vt2)=x2(1−m+η+2m+ηΦ(ηT)−2e−μTΦ(m+T)) +xμ2(μ+r^)[1−|m−|η+2|m−|ηΦ(−ηTsign⁡m−)       −2e−(μ+r^)TΦ(−m−T)],

where *η* ≡ *m*_+_^2^ + 2*μ* and *m*_±_ are defined above (cf. (13)).

Thus, the full premium for a contract paying Ψ_∂_ = max⁡{*X*_∂_, *X*_*t*_ *e* ^*δ*(∂−*t*)^} at the policyholder's demise (∂ = *τ*) or else at expiry of the policy (∂ = *T*) is given by

(18) vt≡T^pd+tvt1)+vt2)=x2(1−m+η+2m+ηΦ(ηT)) +xμ2(μ+r^)[1−|m−|η+2|m−|ηΦ(−ηTsign⁡m−)       +2r^e−(μ+r^)TΦ(−m−T^)].

The premium (17) simplifies when the insurance company is committed to pay a technical interest rate *δ* equal to the short rate *r*. In this case, *m*_+_ = −*m*_−_ = *σ*/2, *η* = (*σ* ^2^/4) + 2*μ*, and (17) yields

(19) $$\begin{array}{c} v_{t}^{2)}=x\left[1-\frac{\sigma }{2\sqrt{\eta }}+\frac{\sigma }{\sqrt{\eta }}\Phi \left(\sqrt{\eta \hat{T}}\right)-2e^{-\mu \hat{T}}\Phi \left(\frac{\sigma }{2}\sqrt{\hat{T}}\right)\right]. \end{array}$$

**Figure 1:**
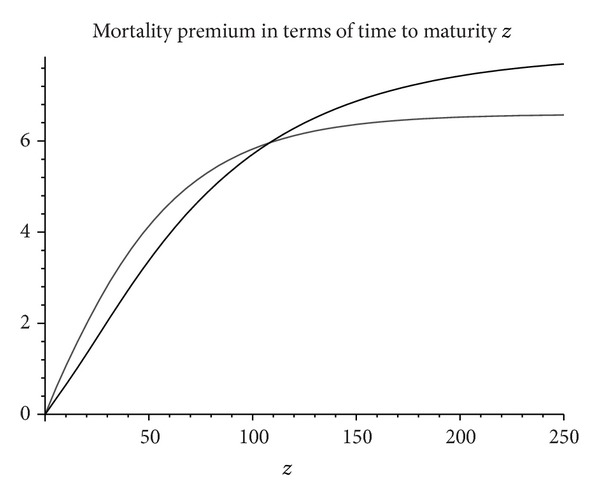
Mortality premium as a function of time to maturity given in years corresponding to a constant annual interest rate *r* = 4.5%. Other parameters are *x* = 5,  *σ* = 25%, *μ* = 0.015 (thick solid line) and *x* = 5, *σ* = 15%, *μ* = 0.025 (thin line).

In Figure 1 we plot *v* ^2)^(*T* | *t*, *x*) as a function of *T* − *t*. As expected, the premium vanishes if *μ* = 0. Otherwise it increases towards $x(1+\sigma /2\sqrt{\eta })$ as the graph shows.

Further, the corresponding full premium (18) can be written in a quite neat way as

(20) $$\begin{array}{c} v_{t}\equiv v_{t}^{1)}+v_{t}^{2)}=x\left[1+\frac{\sigma }{\sqrt{\eta }}\left(\Phi \left(\sqrt{\eta \hat{T}}\right)-\frac{1}{2}\right)\right]. \end{array}$$

### 3.2. Floating Strike Lookback Options

#### 3.2.1. Maturity Component (Ψ_*T*_^1)^  =  max⁡_0≤*s*≤*T*_ *X*_*s*_)

European floating strike lookback options are a different interesting kind of GULC. For a * put option* (flop) they entitle the owner to sell the stock at the highest realized price; thus, they imply a terminal payoff Ψ_∂_ = **X**_∂_^*t*^ − *X*_∂_, where **X**_*T*_^*t*^ ≡ max⁡_*t*≤*s*≤*T*_ *X*_*s*_ is the running maxima of the process. Lookback options guarantee a nonloss outcome and as such are interesting in dynamic investment fund protection.

Let *W*_*t*_^*v*^ be BM with drift *v* and let $M_{\hat{T}}\equiv \max_{t\leq s\leq T}W_{s}^{v}$ be the running maxima. The distribution of the latter can be obtained in terms of the joint density of BM without drift and its running maximum as follows.

For standard BM, *W*_*t*_ is obviously **W**_*T*_^*t*^ ≡ max⁡_*t*≤*s*≤*T*_ *W*_*s*_ = **W**_*T*−*t*_^0^ ≡ **W**_*T*−*t*_. If (*W*_*T*_^*v*^, **W**_*T*_^*v*^) are BM with drift rate *v* and its running maxima, their joint density may be retrieved by the known [18] joint density of *W*_*t*_ (BM without drift) and **W**_*T*_, the Radon-Nikodym transformation $d\mathbb{P}=\exp(vW_{\hat{T}}^{v}-(v^{2}/2)\hat{T})d\mathbb{Q}$ along with the Cameron-Martin-Girsanov theorem. One finds, upon evaluation of some integrals, that

(21) $$\begin{array}{c} {𝔼}^{\mathbb{P}}\left(1_{W_{T}^{v}<y}\right)={𝔼}^{\mathbb{Q}}\left(e^{vW_{\hat{T}}^{v}-(v^{2}/2)\hat{T}}1_{M_{\hat{T}}<y}\right)=\overset{y}{\underset{0}{\int }}q\left(\hat{T},y\right)dy, \end{array}$$

where

(22) q(T^,y)=12πT^e−(y−vT^)2/2T^ +exp⁡[2vy](12πT^e−(y+vT^)2/2T^−2vΦ(−y−vT^T^)),                     y≥0.

The distribution of the running maxima of GBM follows from that of the running maxima of BM with drift $M_{\hat{T}}$ by noting that if *X*_*s*_^*t*,*x*^, *t* < *s* is the solution to (1) with drift *r* passing through *x* at time *t* then

(23) Xs≡Xs0,x0=Xst,Xt0,x0=Xst,x=Xtexp⁡σ(Ws−Wt+vs^)=Lawϱ(Ws^v),

where $\hat{s}\equiv s-t$, *ϱ*(*z*) = *xe* ^*σz*^ is increasing and *W* ^*v*^ is a BM with drift *v* ≡ *r*/*σ* − *σ*/2. It follows that

(24) $$\begin{array}{c} \underset{t\leq s\leq T}{\max}X_{s}\overset{\text{Law}}{=}\underset{t\leq s\leq T}{\max}\varrho \left(W_{s}^{v}\right)=\varrho \left(\underset{t\leq s\leq T}{\max}W_{s}^{v}\right). \end{array}$$

Therefore,

(25) $$\begin{array}{c} {𝔼}^{\mathbb{P}}\left(\underset{t\leq s\leq T}{\max}X_{s}\;|\;X_{t}=x\right)={𝔼}^{\mathbb{P}}\left(xe^{\sigma M_{\hat{T}}}\right)=\overset{\infty }{\underset{0}{\int }}xe^{\sigma y}q\left(\hat{T},y\right)dy. \end{array}$$

By using (22) and upon tedious integration we obtain that the the premium for a flop contract written at *t* = 0 and maturity at *T* is given by

**Figure 2:**
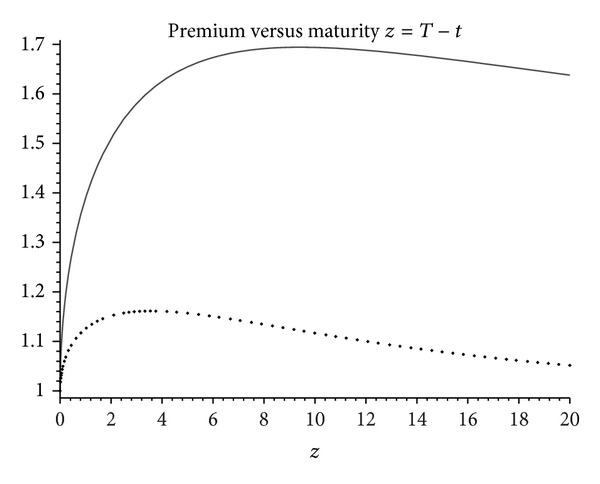
Maturity component premium as a function of time to maturity in years corresponding to benefits Ψ_*T*_^1)^ = max⁡{*X*_*T*_, *x*} and Ψ_*T*_^1)^ = max⁡_0≤*s*≤*T*_ *X*_*s*_. The thick dark line is the max-premium (26), whereas (12) is the thin, lower line. Parameters are taken as *x* = 1, *r* = 0.045, and *σ* = 20%.

(26) vt1)=T^pd+tx((1+σ22r)Φ(m+T)     +(1−σ22r)e−rTΦ(m−T)),

where we define now *m*_±_ ≡ *r*/*σ* ± *σ*/2 (note that they coincide with (13) when *δ* = 0). In Figure 2 we compare this premium with that of the Brennan-Schwartz-Boyle formula (12) corresponding to the case *δ* = 0. Notice how (26) is significantly larger than the premium (12). The humped form is in this case characteristic of the latter but not necessarily of the max-premium.

#### 3.2.2. Floating Strike Options: Demise Component (Ψ_*τ*_^2)^  =  max⁡_0≤*t*≤*τ*_ *X*_*s*_)

We now evaluate the demise component to the premium for a Flop contract written at *t* = 0 when the hazard function *μ* is time independent. Here Ψ_*τ*_^2)^ = max⁡_0≤*t*≤*τ*_ *X*_*s*_ and it follows from (10) that

(27) vt2)≡∫0Th(s)Bs𝔼xℙΨs2)ds=μx∫0Tdse−μs(1+σ22r)Φ(m+s) +(1−σ22r)e−(μ+r)sΦ(m−s),

where we recall that *m*_±_ ≡ *r*/*σ* ± *σ*/2, *η* = *m*_+_^2^ + 2*μ*. By recalling (16) we obtain the fair premium as

(28) vt2)=σm+x2r(1−m+η+2m+ηΦ(ηT^)−2e−μT^Φ(m+T^)) +σm−xμ2r(μ+r)[1−|m−|η+2|m−|ηΦ(ηT^sign⁡m−)        −2e−(μ+r^)T^Φ(m−T^)].

## 4. Price Dynamics When Supports Are Present

Here we describe a model of price dynamics that incorporate the possibility of the existence of a strong lower support at some constant level *c*, where 0 < *c* < *x*_0_ ≡ *X*_0_. To meet this requirement one must modify appropriately the stock dynamics. We assume the existence of a risk neutral probability ℙ* and a process *W*_t_* which is a BM with respect to the latter such that the risk neutral evolution of *X*_*t*_ satisfies the SDE:

(29) $$\begin{array}{c} dX_{t}=r_{t}X_{t}dt+b\left(t,X_{t}\right)dW_{t}^{*},\;\;X_{0}=x_{0}>c, \end{array}$$

where the infinitesimal variance coefficient *b*(*t*, *x*) is to be defined appropriately.

**Remark 1**

Valuation under exotic dynamics where the stock is driven by a SDE whose variance coefficient *b*(*t*, *x*) is not a linear function of *x* has often been the subject of financial literature, even as early as 1976. A familiar case is the CEV model of Cox [19], where *b*(*x*) = *σx* ^*ρ*^ and *σ* and *ρ* < 1 are constants. A more complete account of prizing under these dynamics is given in Delbaen and Shirakawa [20]. The extension of CEV models to *b*(*x*) having also jump singularities is discussed by Decamps et al. [21]. Our stochastic evolution equation (see (29)) is reminiscent, to some extent, of models describing stochastic interest rate dynamics, such as the classical CIR model [22] where the underlying is related to a Bessel process. For other models in this regard see Schroder [23], Geman and Yor [24], and Goovaerts and de Schepper [25]. Pricing of equity linked products with the stock following some exotic dynamics driven by Levy processes appears in Jaimungal and Young [26].

We find it reasonable to assume that there exists positive probability to attain the boundary; we suppose that this event “triggers” bid orders and hence that *X*_*t*_ ricochets upon hitting the boundary. This requirement yields that the point *x* = *c* must be what, in the terminology of Feller's boundaries classification, is termed a regular boundary. We also require that for large *x*, *b*(*t*, *x*) displays a linear dependence on *x*, as happens with the GBM (1). The obvious choice *b*(*t*, *x*) = *σ*(*x* − *c*) is not acceptable since under such dynamics *c* is a natural, nonattainable barrier. The simplest choice that meets all these requirements is given by taking $b(t,x)=\sigma (t)\sqrt{x^{2}-c^{2}}$, where *σ*(*t*) is an arbitrary function; that is,

(30) $$\begin{array}{c} dX_{t}=r_{t}X_{t}dt+\sigma \left(t\right)\sqrt{X_{t}^{2}-c^{2}}dW_{t}^{*},\;\;X_{0}=x_{0}>c. \end{array}$$

Note first that under the risk neutral probability ℙ* the deflated process *X*_*t*_ *e* ^−∫_0_^*t*^*r*_*s*_*ds*^ is a martingale. For ease of notation in the sequel we drop the symbol *. Note also that the square root branch point *x* = *c* prevents *X*_*t*_ to reach the region [0, *c*) but nevertheless there exists positive probability to attain the barrier *x* = *c*.

When *r* and *σ* are constants these ideas are substantiated by appealing to Feller's theory. Let *τ*_*c*_ be the first hitting time of the barrier. The behaviour of the Feller functions depends on the parameter *ϱ* ≡ *r*/*σ* ^2^. The classical scale and speed functions *s*(*x*), *m*(*x*) are given by

(31) $$\begin{array}{c} s\left(x\right)=\overset{x}{\underset{}{\int }}{\left(y^{2}-c^{2}\right)}^{-\varrho }dy,\\ \frac{\sigma^{2}}{2}m\left(x\right)=\overset{x}{\underset{}{\int }}{\left(y^{2}-c^{2}\right)}^{\varrho -1}dy. \end{array}$$

We also consider the Feller functions

(32) $$\begin{array}{c} \Sigma \left(c,x\right)\equiv \overset{x}{\underset{c}{\int }}dm\left(y\right)\left(s\left(y\right)-s\left(c\right)\right),\\ \Omega \left(c,x\right)\equiv \overset{x}{\underset{c}{\int }}dm\left(y\right)\left(s\left(x\right)-s\left(y\right)\right). \end{array}$$

We see that *m*(*c*) and *Ω*(*c*, *x*) are always finite; by contrast *s*(*c*) is finite ⇔Σ(*c*, *c*) is finite ⇔*ϱ* < 1. Thus if *ϱ* < 1 the point *c* is a regular boundary. By Feller's test

(33) $$\begin{array}{c} {\mathbb{P}}_{x_{0}}\left(\tau_{c}\wedge \tau_{\infty }<\infty \right)>0⟺\varrho <1. \end{array}$$

Actually, exploiting further Feller's test we see that

(34) $$\begin{array}{c} {\mathbb{P}}_{x_{0}}\left(\tau_{c}\wedge \tau_{\infty }<\infty \right)=1⟺\Omega \left(c,c\right)<\infty ,\\ s\left(\infty \right)=\infty ⟺\varrho \leq \frac{1}{2}. \end{array}$$

We get a sharper result by noting that if *ϱ* ≤ 1/2 then *s*(*∞*) = *∞* which implies *τ*_*∞*_ = *∞*; thus, Feller's test yields *τ*_*c*_ < *∞* almost surely *ℙ*_*x*_0__ whenever *ϱ* ≤ 1/2.

To determine whether *τ*_*c*_ < *τ*_*∞*_ occurs note that for any *y* > *x*_0_ *ℙ*_*x*_0__(*τ*_*c*_ < *τ*_*y*_) = (*s*(*y*) − *s*(*x*_0_))/(*s*(*y*) − *s*(*c*)). Thus letting *y*↑*∞* we have

(35) $$\begin{array}{c} {\mathbb{P}}_{x_{0}}\left(\tau_{c}<\tau_{\infty }\right)\equiv {\mathbb{P}}_{x_{0}}\left(X_{t}\;\;\text{ever}\;\;\text{reaches}\;\;c\right)=\frac{s\left(\infty \right)-s\left(x_{0}\right)}{s\left(\infty \right)-s\left(c\right)} \end{array}$$

and we conclude that if 0 < *ϱ* < 1 there is positive probability that the support is eventually reached.

We have just seen that if *ϱ* ≤ 1/2 then *τ*_*c*_ < *∞* almost surely *ℙ*_*x*_0__. However even in this case the mean time may be infinite. We can gain some additional information by noting that if *s*(*∞*) = *∞* then

(36) $$\begin{array}{c} {𝔼}_{x_{0}}\left(\tau_{\infty }\right)<\infty ⟺m\left(\infty \right)<\infty . \end{array}$$

We can use this to conclude that *𝔼*_*x*_0__(*τ*_*∞*_) = *∞* if *ϱ* ≥ 1/2 and

(37) σ22𝔼x0(τ∞) =∫cx0(y2−c2)ϱ−1dy∫y∞(z2−c2)ϱ−1dz if  ϱ<12.

The case *σ* ^2^ = 2*r* is of particular interest since then all Feller functions can be evaluated explicitly as

(38) $$\begin{array}{c} s\left(x\right)=\frac{\sigma^{2}}{2}m\left(x\right)=\log\left(\frac{x+\sqrt{x^{2}-c^{2}}}{c}\right),\\ \Sigma \left(c,x\right)=\Omega \left(c,x\right)=\frac{s^{2}\left(x\right)}{\sigma^{2}}. \end{array}$$

Here *s* and *m* are the scale and speed functions. Thus, inasmuch as *c* > 0 we have Σ(*c*, *x*) = *Ω*(*c*, *x*) < *∞* corresponding to a regular boundary.

We now consider the solution of the SDE (29). For general election of the functions *r*(*t*) and *σ*(*t*) the solution remains unknown. However, in the particular case when the time dependent volatility and interest rate satisfy *σ* ^2^(*t*) = 2*r*(*t*), then it turns out that the equation admits an analytical solution; we restrict to this situation in the sequel. Indeed, by using Itô's rule, one can prove that a strong solution to (29) satisfying *X*_0_ = *x*_0_ is given explicitly by

(39) $$\begin{array}{c} X_{t}=c\cosh\left(\overset{t}{\underset{0}{\int }}\sqrt{2r_{s}}dW_{s}+\nu \right), \end{array}$$

where we set

(40) $$\begin{array}{c} \nu \equiv \cosh^{-1}\left(\frac{x_{0}}{c}\right),\;\;\varphi \left(t\right)\equiv 2\log B_{t}=2\overset{t}{\underset{0}{\int }}r_{l}dl,\\ \varphi \left(t,T\right)\equiv \varphi \left(T\right)-\varphi \left(t\right)=2\overset{T}{\underset{t}{\int }}r_{l}dl. \end{array}$$

Further in terms of a new Brownian motion ${\tilde{W}}_{t}$ we have that a weak solution is given by

(41) $$\begin{array}{c} X_{t}=c\cosh Y_{t}\;\text{where}\;\;Y_{t}=\nu +{\tilde{W}}_{\varphi (t)}. \end{array}$$

This follows by noting that $\int_{0}^{t}\sqrt{2r_{s}}dW_{s}$ is a local martingale and Levy's representation theorem.

**Figure 3:**
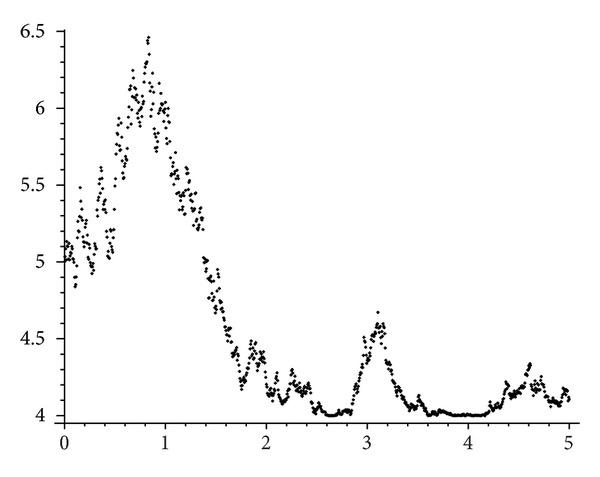
A simulated path of the price process. We plot *X*_*t*_ as a function of time during a time span of five years (*t* = 5). The parameters have been chosen as: *r* = 4.5% yr^−1^, *σ* = 30%, *c* = 4 and *x*_0_ = 5.

In particular these expressions clarify the behavior of the process upon hitting the boundary: *X*_*t*_ attains the barrier *c* whenever the process *Y*_*t*_ reaches 0 upon which *X*_*t*_ is reflected. In Figure 3 a realization of the stock evolution (39) corresponding to a constant interest rate has been plotted. Notice how the stock eventually hits the support level *c* = 4 several times.

Explicit valuation formulae under dynamics (see (39)–(42)) could prove quite awkward to obtain. Here we determine the fair premium under these dynamics for several pure endowment contracts, with general deterministic interest rate *r*_*t*_.

### 4.1. GULC of Type (i) ($\Psi_{T}^{1)}\;=\;\max\{X_{T},X_{t}e^{\delta \hat{T}}\}$)

Let *X*_*T*_^*t*,*x*^, *t* < *T* be the solution to (29) at time *T* that starts from *x* at time *t*. We have

(42) $$\begin{array}{c} X_{T}^{0,x_{0}}=X_{T}^{t,X_{t}^{0,x_{0}}}=c\cosh\left({\tilde{W}}_{\varphi (t,T)}+\varsigma \right), \end{array}$$

where we recall that

(43) $$\begin{array}{c} \varsigma \equiv \cosh^{-1}\left(\frac{x}{c}\right),\;\;\varphi \left(t,s\right)\equiv 2\overset{s}{\underset{t}{\int }}r_{l}dl. \end{array}$$

Notice that in the sequel we simply write *φ*(*t*, *T*) ≡ *φ*. It then follows from (9) that whenever the benefit can be written as Ψ_*T*_^1)^ = Ψ_1_(*X*_*T*_) for some Ψ_1_ : ℝ → ℝ, the fair price is given by

(44) vt1)=T^pd+tBtBT𝔼(Ψ1(XTt,x))=T^pd+tBtBT∫dYΨ1(ccosh⁡(Y+ς))2πφe−Y2/2φ.

In our case $\Psi_{1}(X_{T})=\max\{X_{T},X_{t}e^{\delta \hat{T}}\}$ and evaluating the integral (45) we obtain that the fair price for a Brennan-Schwartz-Boyle contract under dynamics (29) is given by

(45) vt1)T^pd+t=x~(Φ(N+)+Φ(M−))+(x−x~)(Φ(N−)+Φ(M+)) +xe(δ−r)T^−(1−Φ(N)−Φ(M)),

where we introduce

(46) $$\begin{array}{c} k\equiv xe^{\delta \hat{T}},\;\;\tilde{x}\equiv \frac{\left(x+\sqrt{x^{2}-c^{2}}\right)}{2},\\ \tilde{k}\equiv \frac{\left(k+\sqrt{k^{2}-c^{2}}\right)}{2},\\ N=\frac{1}{\sqrt{\varphi }}\log\frac{\tilde{x}}{\tilde{k}},\;\;M=-\frac{1}{\sqrt{\varphi }}\log\frac{\tilde{x}\tilde{k}}{c^{2}},\\ N_{\pm }\equiv N\pm \sqrt{\varphi },\;\;M_{\pm }\equiv M\pm \sqrt{\varphi }. \end{array}$$

This expression generalizes the Brennan-Schwartz-Boyle formula (12) to the case when barriers are present. There are interesting differences. Note first that, unlike what happens with (12), *v*_*t*_^1)^ ≡ *v* ^1)^(*T* | *t*, *x*) does not grow linearly with *x*. Further, for long values of $\hat{T}$, *v* ^1)^ tends either to ${\;}_{\hat{T}}p_{d+t}x\equiv v^{1)}(t|t,x)$, to $2_{\hat{T}}p_{d+t}x$, or to *∞* depending on whether $\hat{r}>0$, $\hat{r}=0$, or $\hat{r}<0$. However convergence takes place with a slower rate than what happens when no resistance is present.

**Figure 4:**
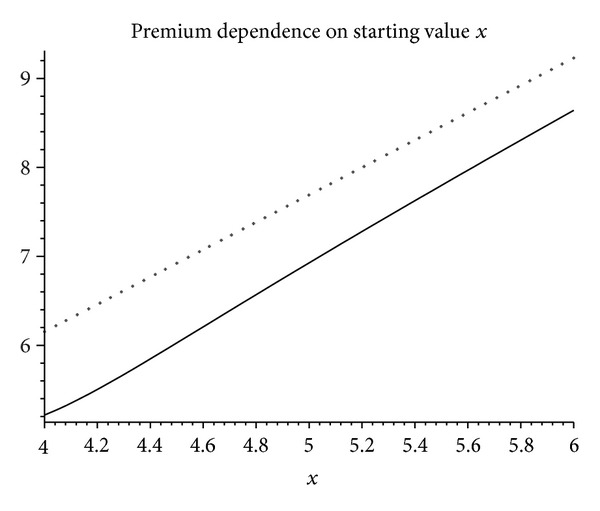
Premium as a function of the initial stock value. Equation (45) is the thick line while (12) is the thin, dotted one. Parameters are chosen as *r* = 4.5% yr^−1^, *σ* = 30%, $\;\hat{T}=6$, and  *δ* = 3.5% yr^−1^.

In Figure 4 we plot the maturity premium *v*_*t*_^1)^ in terms of the initial stock price *x* if the barrier is located at *c* = 4 and compare with the result obtained when no barrier is present. Notice how (12) (dotted line) always overprices the premium compared with (45) given by the thick solid line. This reflects the fact under the actual dynamics that the exposure of the company to a possible plummeting of the stock is partially hedged by the existence of the barrier. For long *x* the difference between both expressions grows dimmer. This is easy to understand, since the protection from the barrier diminishes with the distance to the starting point.

**Figure 5:**
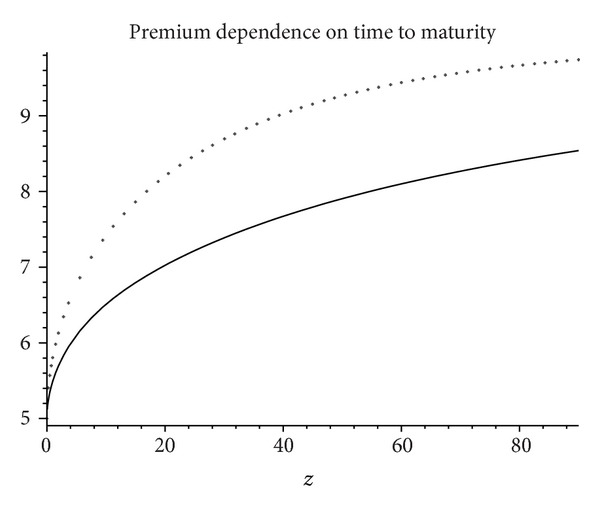


The dependence in time of (45) is displayed in Figure 5. Notice how differences between (12) and (45) can be quite marked for moderate maturity times.

Setting *c* = 0 amounts to having no barrier. In this case one has $\tilde{x}=x$, $\tilde{k}=xe^{\delta \hat{T}}$, and *M* = −*∞*; most of the terms in (45) drop out and, as expected, we recover (12).

### 4.2. GULC of Type (ii) (Ψ_*T*_^1)^  =  max⁡_*t*≤*s*≤*T*_ *X*_*s*_)

We next consider a contract linked to the evolution of the maximum value of the stock, that is, wherein Ψ_*T*_^1)^ = max⁡_*t*≤*s*≤*T*_ *X*_*s*_. Accordingly, the fair price of such contract involves the distribution of $\max_{t\leq s\leq T}\cosh(\varsigma +\int_{t}^{s}\sqrt{2r_{l}}dW_{l})$. This entails important difficulties to derive the fair prize accountable to the fact that under dynamics (29), (24) does not hold since *ϱ*(*z*) ≡ *c*cosh⁡(*z* + *ς*) is not increasing. The max distribution can be given in terms of the survival probability for Brownian motion as we now show. Let *M*_*t*_ and *m*_*t*_ denote respectively running maximum and minimum of BM; then, from (41) one can prove the following equality in law:

(47) max⁡t≤s≤Tcosh⁡(W~φ(t,s)+ς)  =Lawcosh⁡(Mφ(t,T)+ς)1{Mφ(t,T)+mφ(t,T)≥−2ς}    +cosh⁡(mφ(t,T)+ς)1{Mφ(t,T)+mφ(t,T)<−2ς}.

Therefore we have in terms of the joint density *f*(*z*, *y*; *t*) of (*m*_*t*_, *M*_*t*_) that (see (41)–(43))

(48) $$\begin{array}{c} {𝔼}^{\mathbb{P}}\left(\underset{t\leq s\leq T}{\max}X_{s}\;|\;X_{t}=x\right)=c\left(I_{1}+I_{2}\right), \end{array}$$

where

(49) I1≡∫0∞cosh⁡(y+ς)dy∫−2ς−y0f(z,y;φ)dz,I2≡∫0∞dy∫−∞−2ς−ydzf(z,y;φ)cosh⁡(z+ς).

Recall that the survival function *G*(*z*, *y*; *t*), or probability that the BM *W*_*t*_ has remained within the interval [*z*, *y*] up to time *t*, is given by (see [27] or [28]):

(50) G(z,y;t)≡ℙ(z≤mt≤Mt≤y)=∑n=−∞∞Φ(an1t)−Φ(an2t) −Φ(an3t)+Φ(an4t),

where

(51) an1≡(2n+1)y−2nz,an2≡(2n+1)y−2(n+1)z,an3≡2ny−(2n−1)z,an4≡2ny−(2n+1)z.

By differentiation the joint density *f* of (*m*_*t*_, *M*_*t*_) follows. One could proceed by substituting this *f* into (49). However this yields a quite messy expression. A more convenient approach is to write (49) in terms of *G* as

(52) I1=∫0∞cosh⁡(y+ς)(∂yG|z=−2ς−y,y−∂yG|0,y)dy,I2=∫0∞dy∫−∞−2ς−ydzf(z,y;φ)cosh⁡(z+ς)=∫−∞−2ςdzcosh⁡(z+ς)∫0−2ς−zdyf(z,y;φ)=∫−∞−2ςcosh⁡(z+ς)(∂zG|z,0−∂zG|z,−2ς−z)dz.

Note that (50) implies that ∂_*y*_ *G*|_0,*y*_ = ∂_*z*_ *G*|_*z*,0_ = 0. Hence upon appropriate manipulations we find that both terms *I*_1_ and *I*_2_ satisfy

(53) $$\begin{array}{c} I_{1}=I_{2}=\overset{\infty }{\underset{\varsigma }{\int }}dy\cosh y\partial_{y}G\left(-\varsigma -y,y-\varsigma ;\varphi \right). \end{array}$$

This observation allows us to simplify the conditional expectation to

(54) 𝔼ℙ(max⁡t≤s≤TXs ∣ Xt=x)=2c∑n=−∞∞∫ς∞dycosh⁡y((2n+1)pφ(yn+)+2npφ(yn−)           −(2n−1)pφ(y~n+)−2npφ(y~n−)),

where *p*_*t*_(·) is BM density and we introduce

(55) $$\begin{array}{c} \alpha_{n}\equiv 4n+1,\;\;{\tilde{\alpha }}_{n}\equiv 4n-1,\\ y_{n}^{\pm }\equiv \alpha_{n}y\pm \varsigma ,\;\;{\tilde{y}}_{n}^{\pm }\equiv {\tilde{\alpha }}_{n}y\pm \varsigma . \end{array}$$

A further simplification is obtained noting that $y_{n}^{+}=-{\tilde{y}}_{-n}^{-}$ whereupon we have

(56) 𝔼ℙ(max⁡t≤s≤TXs ∣ Xt=x)  =2c∑n=−∞∞αn∫ς∞dycosh⁡y(pφ(yn+)+pφ(yn−)).

The latter integral can be evaluated in terms of the error function Φ; then, upon substitution into (9), we obtain that if *σ* ^2^ = 2*r*_*t*_ and the short rate *r*_*t*_ depends on time in an arbitrary way, then the value of the fair premium is given by

(57) vt1)=T^pd+tc∑n=−∞∞eφ(1−αn2)/2αn2 ×∑ϵ=−1,1 ∑κ=−1,1sne−(ϵκς/αn)Φ(snλnϵ,κ),

where

(58) $$\begin{array}{c} s_{n}\equiv \mathrm{sign}\alpha_{n},{\;\;\lambda }_{n}^{\epsilon ,\kappa }\equiv \frac{\epsilon \sqrt{\varphi }}{\alpha_{n}}-\frac{\left(\alpha_{n}+\kappa \right)}{\sqrt{\varphi }}\varsigma . \end{array}$$

The dependence of the premium on time and initial value enters through the variables (49). For a time to maturity ranging from moderate to large only those terms corresponding to *n* = 0 will contribute significantly. It is then natural to write

(59) vt1)=T^pd+tc∑ϵ=−1,1[eϵςΦ(ϵφ)+e−ϵςΦ(ϵφ−2ςφ)         +∑n≠0eφ(1−αn2)/2αn2∑κ=−1,1sne−(ϵκς/αn)Φ(snλnϵ,κ)].

This representation makes it possible to recover (26) when *c* → 0 and *r* is constant. Note that in this case $\varphi =2r\hat{T}$; further, if *n* ≠ 0 then *s*_*n*_ *λ*_*n*_ → −*∞* and Φ(*s*_*n*_ *λ*_*n*_^*ϵ*,*κ*^) → 0. Likewise we have

(60) $$\begin{array}{c} e^{-\varsigma }=\frac{x}{c}-\sqrt{\;{\left(\frac{x}{c}\right)}^{2}-1}⟶0,\\ e^{\varsigma }\Phi \left(\sqrt{\varphi }-\frac{2\varsigma }{\sqrt{\varphi }}\right)\leq e^{-(\varsigma 2/2\varphi )}⟶0,\;\;ce^{\varsigma }⟶2x \end{array}$$

as *c* → 0. Thus, the only nonvanishing term corresponds to *ϵ* = −*κ* = 1, *n* = 0, and

(61) $$\begin{array}{c} v_{t}^{1)}\underset{c\to 0}{⟶}2x_{\hat{T}}p_{d+t}\Phi \left(\sqrt{2r\hat{T}}\right) \end{array}$$

which is (26) in the case $\sigma =\sqrt{2r}$.

## 5. Kolmogorov Backwards Equation for the Premium

In this section we will suppose that the insurer's benefit can be represented as

(62) $$\begin{array}{c} \Psi_{\partial }^{i)}=\Psi_{i}\left(\partial ,X_{\partial }\right)\;\text{where}\;\;\partial =\{\begin{array}{cc} T & \text{if}\;\;i=1,\\ \tau & \text{if}\;\;i=2. \end{array}\; \end{array}$$

Further, we assume that Ψ_*i*_ : ℝ^2^ → ℝ is a piecewise continuous function that does not have a parametric dependence on the “initial value” *x* ≡ *X*_*t*_. Under these assumptions and conditional on {*τ* > *t*} having happened, we next derive a PDE that the premium *v*_*t*_ ≡ *v* (*T* | *t*, *x*) satisfies. Notice that, from (9),

(63) $$\begin{array}{c} \partial_{t}\left(\frac{B_{T}v_{t}^{1)}}{B_{t}}\right)={}_{\hat{T}}p_{d+t}\left[\mu \left(t+d,t\right)\psi \left(t,x\right)+\partial_{t}\psi \left(t,x\right)\right], \end{array}$$

where *ψ*(*t*, *x*) ≡ *𝔼* ^*ℙ*^(Ψ_1_(*T*, *X*_*T*_) | *X*_*t*_ = *x*). Recall that if *X*_*t*_ is a diffusion with drift coefficient *a*(*t*, *x*) ≡ *r*_*t*_ *x* and variance coefficient *b*(*t*, *x*) then the conditional expectation solves Kolmogorov equation:

(64) $$\begin{array}{c} A\psi \left(t,x\right)\equiv \left(\frac{\partial }{\partial t}+a\frac{\partial }{\partial x}+\frac{b^{2}}{2}\frac{\partial^{2}}{\partial x^{2}}\right)\psi \left(t,x\right)=0. \end{array}$$

Operating with the infinitesimal generator **A** on (9) and using (63) we find

(65) $$\begin{array}{c} \left(A-r_{t}-\mu \left(t+d,t\right)\right)v_{t}^{1)}=0. \end{array}$$

Likewise we find that

(66) ∂tvt2)=(r+μ(t+d,t))vt2)−μ(t+d,t)Ψ2(t,x) +∫tTdsh(s)∂t𝔼ℙ(Ψ2(s,Xs) ∣ Xt=x).

Operating again with operator **A** we get

(67) $$\begin{array}{c} \left(A-r_{t}-\mu \left(t+d,t\right)\right)v_{t}^{2)}=-\mu \left(t+d,t\right)\Psi_{2}\left(t,x\right). \end{array}$$

It follows that *v* ≡ *v* ^1)^ + *v* ^2)^ satisfies the backward PDE with final condition

(68) (∂∂t+a∂∂x+b22∂2∂x2−rt−μ(t+d,t))v(T ∣ t,x)  =−μ(t+d,t)Ψ2(t,x),v(T ∣ T,x)=Ψ1(T,x),

where *a*(*t*, *x*) ≡ *r*_*t*_ *x* and $b(t,x)=\sigma (t)\sqrt{x^{2}-c^{2}}$.

**Remark 2**

(1) If *r* and *μ* are constants, there is no resistance: *c* = 0 and, in addition, Ψ_2_(*t*, *x*) = *x*, then (68) reduces to the equation considered by Shen and Xu [12].

(2) If representation (62) holds but Ψ^*i*)^ depends parametrically on the “initial values and times”: Ψ^*i*)^ = Ψ_*i*_(*T*, *X*_*T*_; *t*, *x*), then it does not exist such a clear-cut equationas (68). Notice that this is precisely what happens for the type i GULC considered in this paper. Similarly, the case of path-dependent payoff functions (type ii GULC) may not be covered either with the PDE approach.

## References

[1] Brennan MJ, Schwartz ES (1976). The pricing of equity-linked life insurance policies with an asset value guarantee. *Journal of Financial Economics*.

[2] Boyle PP, Schwartz ES (1977). Equilibrium prices of guarantees under equity-linked contracts. *The Journal of Risk and Insurance*.

[3] Bacinello AR, Ortu F (1993). Pricing equity-linked life insurance with endogenous minimum guarantees. *Insurance: Mathematics and Economics*.

[4] Aase KK, Persson SA (1994). Pricing of unit-linked life insurance policies. *Scandinavian Actuarial Journal*.

[5] Brennan MJ, Schwartz ES (1979). Alternative investment strategies for the issuers of equity linked life insurance policies with an asset value guarantee. *Journal of Business*.

[6] Ekern S, Persson S-A (1996). Exotic unit-linked life insurance contracts. *The GENEVA Papers on Risk and Insurance Theory*.

[7] Boyle PP, Hardy MR (1997). Reserving for maturity guarantees: two approaches. *Insurance: Mathematics and Economics*.

[8] Grosen A, Jorgensen PL (2000). Fair valuation of life insurance liabilities: the impact of interest rate guarantees, surrender options, and bonus policies. *Insurance: Mathematics and Economics*.

[9] Moeller T (2001). Hedging equity-linked life insurance contracts. *North American Actuarial Journal*.

[10] Bernard C, Le Courtois O, Quittard-Pinon F (2005). Market value of life insurance contracts under stochastic interest rates and default risk. *Insurance: Mathematics and Economics*.

[11] Bacinello AR (2005). Endogenous model of surrender conditions in equity-linked life insurance. *Insurance: Mathematics and Economics*.

[12] Shen W, Xu H (2005). The valuation of unit-linked policies with or without surrender options. *Insurance: Mathematics and Economics*.

[13] Black F, Scholes M (1973). The pricing of options and corporate liabilities. *Journal of Political Economy*.

[14] Merton RC (1973). Theory of rational option pricing. *The Bell Journal of Economics and Management Science*.

[15] Marocco P, Pitacco E Longevity risk and life annuity reinsurance.

[16] Melnikov A, Romaniuk Y (2006). Evaluating the performance of Gompertz, Makeham and Lee-Carter mortality models for risk management with unit-linked contracts. *Insurance: Mathematics and Economics*.

[17] Harrison JM, Pliska SR (1981). Martingales and stochastic integrals in the theory of continuous trading. *Stochastic Processes and their Applications*.

[18] Levy P (1948). *Processus Stochastiques et Movement Brownien*.

[19] Cox JC (1996). The constant elasticity of variance option pricing model. *Journal of Portfolio Management*.

[20] Delbaen F, Shirakawa H (2002). A note on option pricing for the constant elasticity of variance model. *Asia-Pacific Financial Markets*.

[21] Decamps M, de Schepper A, Goovaerts M (2004). Applications of *δ*-function perturbation to the pricing of derivative securities. *Physica A*.

[22] Cox J, Ingersoll J, Ross S (1985). A theory of the term structure of interest rates. *Econometrica*.

[23] Schroder M (1989). Computing the constant elasticity of variance option pricing. *Journal of Finance*.

[24] Geman H, Yor M (1993). Bessel processes, Asian options and perpetuities. *Mathematical Finance*.

[25] Goovaerts M, de Schepper A (1997). IBNR reserves under stochastic interest rates. *Insurance: Mathematics and Economics*.

[26] Jaimungal S, Young VR (2005). Pricing equity-linked pure endowments with risky assets that follow Lévy processes. *Insurance: Mathematics and Economics*.

[27] Freedman D (1971). *Brownian Motion and Difussions*.

[28] Revuz D, Yor M (1991). *Continuous Martingales and Brownian Motion*.

